# Gestational age-related patterns of *AMOT* methylation are revealed in preterm infant endothelial progenitors

**DOI:** 10.1371/journal.pone.0186321

**Published:** 2017-10-16

**Authors:** Giovanna Vinci, Christophe Buffat, Stéphanie Simoncini, Farid Boubred, Isabelle Ligi, Florent Dumont, Bernard Le Bonniec, Thierry Fournier, Daniel Vaiman, Françoise Dignat-George, Umberto Simeoni

**Affiliations:** 1 Cochin Institute, Inserm U1016, CNRS 8104, Université Paris Descartes, 27 Rue du Faubourg Saint-Jacques, Paris, France; 2 UMR-S1139 Inserm, Université Paris Descartes, Faculté de Pharmacie, Paris, France; 3 Department of Neonatology Hôpital La Conception, 147 Boulevard Baille, Marseille, France; 4 UMR 1076 INSERM, Aix-Marseille Université, 27 Boulevard Jean Moulin, Marseille, France; 5 IPSIT—Institut Paris-Saclay d'Innovation Thérapeutique UPSud—UFR Pharmacie, 5 rue J.B. Clément, Châtenay-Malabry, France; 6 UMR_S1140 Inserm, Université Paris Descartes; Faculté de Pharmacie, Paris, France; 7 Division of Pediatrics and DOHaD Laboratory, CHUV and Université de Lausanne, rue du Bugnon 46, Lausanne, Switzerland; University of Southampton, UNITED KINGDOM

## Abstract

**Objective:**

Preterm birth is associated with altered angiogenesis and with increased risk of cardiovascular dysfunction and hypertension at adulthood. We previously demonstrated that in preterm newborns circulating cord blood endothelial progenitor cells (ECFC), responsible for angio/vasculogenesis, are reduced in number and display altered angiogenic properties. Altered angiogenic function was associated with a decreased expression of pro-angiogenic genes, among which the *AMOT* gene which is a strong positive regulator of angiogenesis. Such dysregulation may be related to epigenetic factors. In this study we analyse the methylation profiling of the *AMOT* gene during development, through a comparative analysis of the cord blood ECFC of preterm newborns and their term counterpart.

**Methods:**

We used both cloning-sequencing and pyrosequencing experiments to perform a comparative analysis of the DNA methylation profile of the promoter CpG island of *AMOT* gene in the cord blood ECFC of 16 preterm newborns (28–35 weeks gestational age-GA) and 15 term newborns (>37 weeks GA).

**Results:**

Twenty nine clones (obtained from 2 term newborns) and forty clones (obtained from 3 preterm newborns) were sequenced. The *AMOT* gene methylation rate was significantly higher in preterm compared to term newborns (4.5% versus 2.5% respectively: *χ2 =* 3.84; P = 1.8 10^−02^). Bisulfite pyrosequencing identified four CpG dinucleotides with significantly higher methylation levels in preterm newborns. This CpG-targeted methylation significantly decreased with increasing gestational age.

**Conclusions:**

These findings highlight importance of pro-angiogenic *AMOT* gene methylation in ECFC, suggesting that epigenetic mechanisms may control the regulation of angiogenesis during development. Therefore they pave the way to specific short term and long term complications of preterm birth by altered angiogenesis.

## Introduction

Preterm birth is associated with an increased risk of cardiovascular dysfunction and hypertension at adulthood, with a clear correlation to the degree of immaturity [1–3]. Epidemiologic, clinical and animal studies have shown that the period extending from conception to early infancy constitutes a particular window of vulnerability for the developmental programming of later cardio-vascular function and the risk for diseases such as stroke, type 2 diabetes, obesity and other non-communicable diseases at adulthood [4,5]. The concept of « developmental programming » results from such observations and proposes that stimuli that occur during this period, such as nutritional changes or exposure to stress, influence the set point of physiologic regulations and may thus predispose to health or disease susceptibility in adulthood [6].

The epigenome is sensitive to environment cues during development. In particular, the interaction of genetic and environmental factors during the periconceptional, fetal and early infantile periods influences the risk for hypertension [7,8]. Such effect is likely mediated through epigenetic changes that cause the long term phenotype of an individual and are inheritable, leading to intergenerational effects [9].

Multiple mechanisms contribute to the early programming of hypertension, involving the kidney [10], the neuroendocrine system [11], and the vasculature, through vascular/endothelial dysfunction and altered capacity of angiogenesis/vasculogenesis and of vascular repair by circulating endothelial progenitor cells [12–16]. Indeed, the number of the endothelial progenitor cells ECFC (endothelial colony-forming cells), which are responsible for angiogenesis, is lower in very preterm infants than in term controls [17,18].

We previously showed that in preterm newborns the cord blood ECFC are reduced in number and their angiogenic potential *in vitro* is impaired. The expression of genes with anti-angiogenic functions is up-regulated, while the expression of *AMOT* which is a key gene in the control of angiogenesis [19], as well as of other pro-angiogenic genes, is reduced [18].

Recent data underline the crucial role of *AMOT* in angiogenesis. *AMOT* knockout mice show a high embryonic lethality (75%, around embryonic day 11), with severe vascular insufficiency in the intersomitic region and dilated vessels in the brain (19). However, little information is available on the early epigenetic modifications of *AMOT*, as well as of other angiogenic genes, in preterm birth which is associated with the risk of developmental programming of hypertension.

Here we designed experiments to compare the DNA methylation profile of the CpG island of *AMOT* gene promoter in cord blood ECFC from preterm newborns and from their term counterpart. The results demonstrate higher rates of *AMOT* methylation in preterm infants. Furthermore, the methylation level is inversely correlated to gestational age. Given the role of vascular-endothelial dysfunction in the mechanisms of developmentally programmed hypertension, these findings suggest that the pro-angiogenic gene *AMOT* is involved and may have an important role in the development of the vascular system.

## Patients and methods

### Patients

Fifteen term and sixteen preterm newborns were studied.

Inclusion criteria were: gestational age (GA) ranging from 28 to 35 weeks (premature newborns) or more than 37 weeks GA (term newborns), estimated by early antenatal ultrasounds and/or the date of the last menstrual period.

Neonatal exclusion criteria were: viral infections, major congenital heart malformations, genetic abnormalities, structural brain malformations and metabolic diseases.

Maternal exclusion criteria were: - unstable maternal non-psychiatric medical illnesses requiring pharmacological treatment during pregnancy (e.g., asthma, autoimmune disorders);—abnormal maternal thyroid stimulating hormone;—maternal use of lithium, stimulants or migraine medications;—presence of a familial history of cardiovascular disease.

Approval from the Comité de Protection des Personnes Marseille n°2 IRB was obtained. All the parents gave written informed consent for the use of cord blood, in accordance with the Declaration of Helsinki.

### Culture of umbilical cord blood-derived ECFC

ECFC were isolated from the mononuclear cell fraction (MNC) from umbilical cord blood of term (GA >37 weeks) and preterm newborns (GA between 28 and 35 weeks).

MNCs were resuspended in complete EGM2-MV medium, plated at 5x20^6^ cells/well in 6-well plates (0.2% gelatin-coated) and cultured [20]. Cells from passage 5 (P5) were harvested for analysis [18].

### *AMOT* promoter CpG island

**Fig 1:**
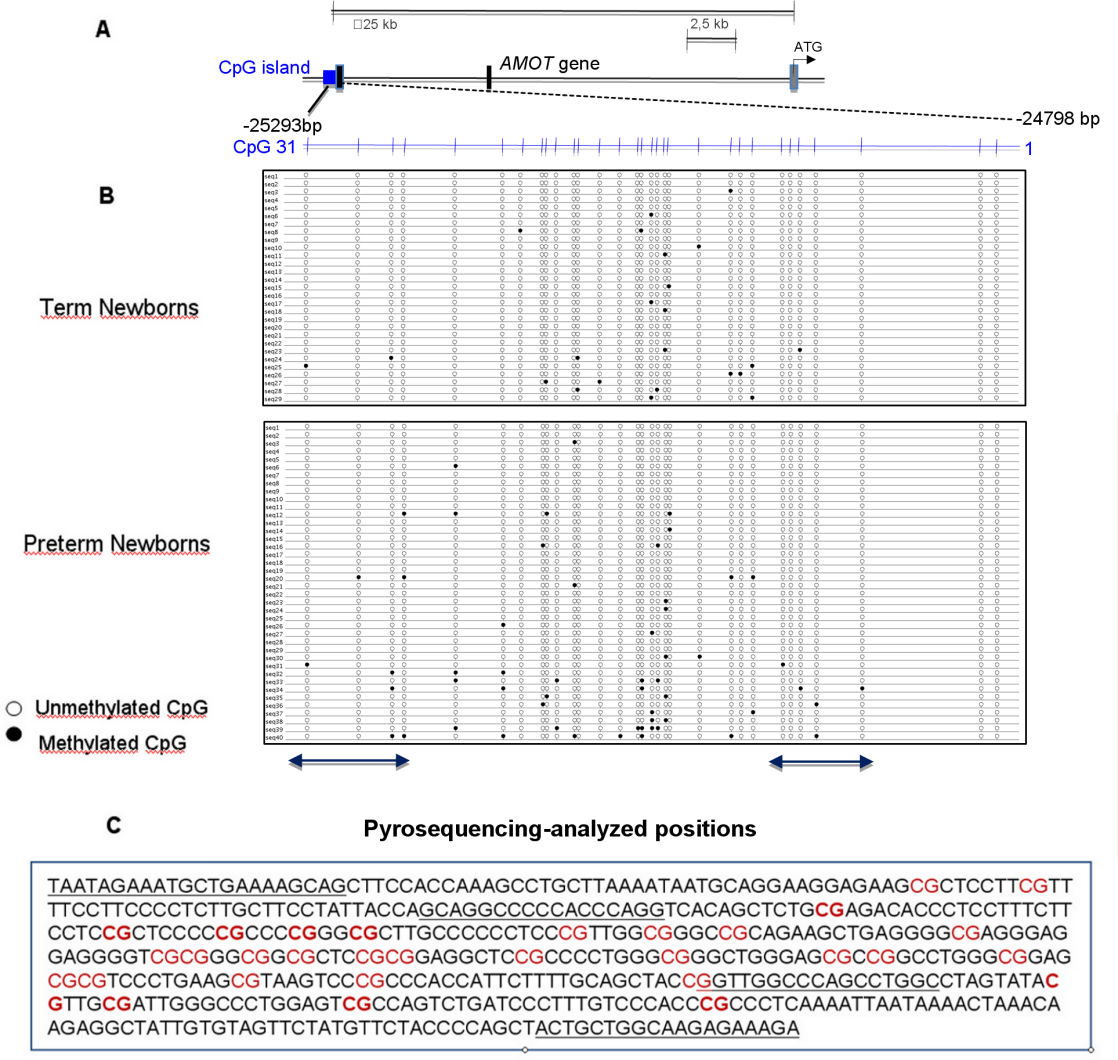
Cloning-sequencing of individual clones of the *AMOT* promoter CpG island. Differences in the methylation of *AMOT* promoter CpG island in newborns are associated with prematurity. (A) Structure of the *AMOT* CpG proximal promoter island region. Structure of the proximal promoter region of the *AMOT* gene showing the location of the CpG island: the methylation CpG sites analysed were numbered 1–31. (B) Bisulfite genomic sequencing of individual clones of the *AMOT* promoter CpG island. Each column represents the methylation status at each CpG dinucleotide in individual clones. Each line represents an individual clone in which the methylation status is determined at each of the 31 CpG dinucleotides. Twenty nine clones from cord blood ECFC of 2 term newborns and forty clones of 3 preterm newborns were examined, respectively. Black and white dots indicate methylated and unmethylated CpGs, respectively. The positions of CpG dinucleotides further analysed by pyrosequencing are indicated by the two lines below dot plots. (C) Sequence of the *AMOT* promoter CpG island. Pyrosequencing primers are underlined. The nine CpG dinucleotides analysed by pyrosequencing are in bold.

The human *AMOT* promoter CpG island is localized in the genomic region at the 5’ extremity of the first exon, at 25293bp from the ATG start codon of the *AMOT* gene (GenBank accession no. NG_022628.2 G2:255522822) (Fig 1A).

### DNA extraction and sodium bisulfite treatment

Genomic DNA was extracted from frozen ECFC samples (FastDNA*®*Kit; MP Biomedicals, LLC) and quantified (Nanodrop-1000, Wilmington, DE). 600 ng of each sample were modified by sodium bisulfite to convert unmethylated cytosines to uracil (EZ DNA Methylation*-*Gold*™* Kit; Zymo Research Corp., Orange, CA, USA). Bisulfite-converted DNA was amplified by PCR using primers corresponding to a region of 495 nucleotides within the *AMOT* promoter CpG island (*AMOT* gene, GenBank accession no. NG_012628.1 G1:255522812).

Specific primer sequences were: Forward primer-TAATAGAAATGTTGAAAAGTAG and Reverse primer-ATCTTTCTCTTACCAACAATA.

PCR reactions were carried out in a final volume of 25 μl using a mixture containing 80 ng of bisulfite-converted DNA, all 4 dNTPs (each at 200 mM), 1.5 mM MgCl2, 1.25 unit of *Platinum*® *Taq* DNA Polymerase (Invitrogen), and each primer at 0.25 mM. The conditions of amplification were: heating at 95°C (5 min), followed by 40 cycles at 95°C (2 min), 54°C (2 min), 72°C (2 min) and ended by a final extension at 72°C (20 min).

### TOPO-TA cloning and sequencing

The PCR products were cloned in a TOPO-TA® vector (pCR*®*2.2*-*TOPO*®;* Invitrogen®). After bacterial transformation by electroporation, the transformed TOPO-TA *E*. *Coli* cells were plated in LB/Amp plates with IPTG/X-gal for blue/white selection of clones. The white clones were PCR-amplified using primers specific of the M13 vector.

Methylation data were obtained in a region containing thirty-one CpG dinucleotides. Samples from both preterm and term newborns were randomly selected. To evaluate CpG methylation throughout the amplified region, twenty nine clones from the cord blood ECFC of two term newborns (>37 weeks GA) and forty clones from three preterm newborns (28 weeks GA) were sequenced (ABI 3700 automated DNA sequencer) using the cloning primers M23F and M23R (sequencing platform of Cochin Institute, Paris).

### DNA methylation by pyrosequencing

The bisulfite-converted DNA from the cord blood ECFC of fourteen term (38–41 weeks GA) and fourteen preterm newborns (28–35 weeks GA), randomly selected, was PCR-amplified and analysed by pyrosequencing. Because we obtained small amounts of DNA, each sample was bisulfite treated only once, but all pyrosequencing assays were performed in duplicates (sometimes in triplicate, but the differences between the results were < 5%).

Primers were specifically designed to read five CpG dinucleotides at the 5’ extremity of the *AMOT* CpG island and four CpG dinucleotides at 3’ extremity:

Forward TAA-TAG-AAA-TGT-TGA-AAA-GTA-G, reverse Biot- ATC-TTT-CTC-TTA-CCA-ACA-ATA, pyrosequencing primers GTA-GGT-TTT-TAT-TTA-GG and GGT-TGG-TTT-AGT-TTG-GT. We used 80 ng of the bisulfite-treated DNA (2 μL) in a 25-μL PCR reaction (Pyromark PCR kit, Qiagen) in which one PCR primer was biotinylated. The Sepharose beads with the immobilized PCR products were purified, denatured (0.2 M NaOH) and washed (PyroMark Q96 Vacuum Prep Workstation, Qiagen). After annealing 10 µL of the 0.3-μmol/L pyrosequencing primer to the purified single-stranded PCR product, pyrosequencing was performed (PyroMark Q96 MD Pyrosequencing System, Qiagen).

Level of DNA methylation was computed as the ratio of C to T peaks for a given CpG site in the target sequence using the PyroQ CpG software (Qiagen). 0%, 50% and 100% methylated DNA were used as standard controls, and a no template control was included in each run, as control for contamination. Each run included standard control DNA: unmethylated (Qiagen, Hilden, Germany), hypermethylated (Millipore, Billerica, MA, USA) and a mixture of both (half methylated). Efficiency of the bisulfite conversion was evaluated through the conversion rate of non-CpG (C-to-T converted was >99%). The PyroQ software includes quality control checkpoints.

The degree of methylation was expressed as the percentage of methylated cytosines over the sum of methylated and unmethylated cytosines.

### Quantitative RT-PCR analysis

Total RNA was extracted (mirVana miRNA Isolation Kit, Ambion) from cord blood ECFC of six preterm and six term neonates randomly selected. Quantitative RT-PCR (qRT-PCR) was performed (Brillant QPCR Master Mix, Stratagene, La Jolla, CA), using pre-designed primers for *AMOT* (Hs00622096_m2) and RPL23 (HS00204273_m2) (Applied Biosystems). Data were generated from each reaction, analysed using Mx3000P software and normalized to the RPL23A RNA. Quantification was performed using the 2-ΔΔ^CT^ method with CT-ECFCs average as the baseline.

### Statistical methods

The nonparametric Mann-Whitney U-test was used in pyrosequencing experiments to analyse each CpG dinucleotide. P*-*values <0.05 were considered statistically significant.

The relationship between the methylation levels for each CpG dinucleotide and gestational age in weeks was then analysed. We applied a linear regression model at every CpG dinucleotide, predicting DNA methylation as a function of gestational age.

## Results

### Clinical data

The clinical characteristics of mothers, term and preterm newborns are described in Table 1.

**Table 1:** Clinical characteristics of patients.

Characteristics	Term Newborns	Preterm Newborns	p
**Maternal data**			
N	15	16	
Age, y	30.9	29.8	NS
Multiple pregnancies, n (%)	0 (0%)	3 (19%)	
Antenatal steroid therapy, n (%)	0 (0%)	14/15 (93%)	< .01
HTA, preeclampsia, n (%)	0 (0%)	3 (19%)	
Premature rupture of membranes, n (%)	0 (0%)	8 (50%)	
Cesarean section, n (%)	11 (73%)	12 (75%)	NS
**Infant data**			
N	15	16	
Mean gestational age, wk	39.6	31.4	< .01
No; by gestational age			
≤ 28 wk	0	1	
28–32 wk	0	9	
32–36 wk	0	6	
≥ 37 wk	15	0	
Mean birth weight, g	3530	1550	< .01
No; with birth weight			
≤ 1500 g	0	9	
1510–2000 g	0	3	
2010–2700 g	1	4	
≥ 2700 g	14	0	
Male	10 (66%)	8 (50%)	NS
Small for gestational age, n (%)	0 (0%)	2 (12%)	

N: number, Y: year, HTA: hypertension, wk: weeks, g: grams, NS: non significant.

A P-value < 0.05 was considered significant.

The patient selection in both groups was only made according to the term or preterm birth; the newborns of the two groups were randomly chosen for the epigenetic and for the expression analysis. Pearson’s Χ squared tests and 2-tailed unpaired t-tests were used for qualitative and quantitative comparisons of variables, respectively. A <0.05 P-value was considered statistically significant.

### Methylation analysis by cloning-sequencing

Methylation data were obtained for every single CpG dinucleotide in the region of the *AMOT* CpG island containing thirty-one CpG dinucleotides. The two newborn populations were randomly selected. Twenty nine clones from two term newborns and forty clones from three preterm newborns containing the specific insert were sequenced. The epigenetic profile showed that some CpG dinucleotides were differentially methylated in the two neonatal populations, with a higher methylation rate in preterm newborns (4.5%) compared to term newborns (2.5%). These differences were statistically significant when assessed as the overall preterm and term newborn population (χ^2^ = 3.84, P = 1.8 10^−02^) (Fig 1A and 1B).

### Methylation analysis by pyrosequencing

To obtain a more precise quantification of cytosine percent methylation, five CpG dinucleotides at the 5’ extremity of the *AMOT* CpG island, and four CpG dinucleotides at the 3’ extremity were analysed by pyrosequencing (Fig 1C). The CpG dinucleotides selected for pyrosequencing have shown a significant higher methylation level in the preterm newborns ECFC in cloning-sequencing experiments described in Fig 1B.

Using the nonparametric Mann-Whitney U-test, three CpG at the 5’ extremity (CpG 5, 6, 7) and one at the 3’ extremity (CpG 28) showed a significantly higher methylation level in preterm newborns (P-values between 0.022 and 7.08E-05) (Table 2).

**Table 2:** Pyrosequencing experiments.

					**Term Newborns**						
**Newborns**	**Sex**	**Weeks (GA)**	**CpG**	**CpG**	**CpG**	**CpG**	**CpG**	**CpG**	**CpG**	**CpG**	**CpG**
			**N3**	**N4**	**N5**	**N6**	**N7**	**N28**	**N29**	**N30**	**N31**
1	M	38	4,50	7,00	5,50	6,00	3,50	8,00	7,50	15,50	8,00
2	M	38	12,33	23,67	10,67	10,67	7,00	6,33	11,00	10,33	14,67
3	M	39	6,00	36,50	7,00	6,00	3,50	3,00	8,00	10,00	8,00
4	M	39	13,00	30,00	12,00	4,00	4,00	11,00	16,00	8,00	16,00
5	M	39	21,00	7,50	9,20	8,10	4,80	9,80	15,20	10,90	9,70
6	F	40	16,50	34,20	8,80	7,50	6,00	7,30	18,10	9,40	9,00
7	M	40	7,00	8,00	6,00	6,00	4,00	7,00	10,00	5,00	11,00
8	M	40	22,50	39,00	6,50	7,50	4,50	8,00	24,00	12,50	29,50
9	M	40	6,00	8,50	7,50	4,50	4,00	4,00	10,50	6,00	12,50
10	F	40	19,00	12,00	7,00	10,00	3,00	10,00	5,00	10,00	19,00
11	F	40	12,50	7,00	4,00	8,00	4,00	8,00	9,00	10,00	7,00
12	F	40	15,00	30,00	10,00	9,00	6,00	5,00	16,00	9,00	20,00
13	M	41	4,50	7,00	5,50	6,00	3,50	8,00	7,50	15,50	8,00
14	M	41	21,00	38,00	10,00	12,00	3,00	9,00	20,00	14,00	20,00
**Mean value**			12,92	20,60	7,83	7,52	4,34	7,46	12,70	10,44	13,74
**S D**			6,51	13,51	2,31	2,31	1,21	2,27	5,56	3,12	6,51
				** **	**Preterm Newborns**	** **	** **				
15	M	28	20,00	36,00	14,00	15,00	10,00	9,00	12,00	14,00	20,00
16	F	28	18,80	22,50	14,70	12,70	6,40	6,40	9,10	7,90	11,50
17	F	30	19,00	43,50	26,00	23,00	14,50	21,00	30,50	10,50	15,00
18	F	30	20,50	62,00	30,00	14,50	11,50	13,50	23,50	11,00	18,00
19	F	30	6,00	20,00	36,00	28,00	25,00	13,00	15,00	12,00	17,00
20	F	30	35,00	60,00	23,00	19,00	14,00	15,00	20,00	9,00	14,00
21	F	30	20,00	26,33	15,52	18,33	12,37	15,20	18,50	10,00	16,50
22	F	31	10,94	19,20	9,24	11,34	8,70	7,80	12,10	8,40	13,40
23	M	32	4,67	17,50	11,67	9,50	7,67	9,00	12,33	12,67	19,67
24	F	32	7,67	14,67	8,00	8,33	5,33	7,00	11,67	12,00	18,33
25	M	32	15,34	35,00	22,83	9,61	11,50	9,00	11,30	12,75	17,15
26	M	33	25,00	32,00	28,00	20,00	10,00	20,00	18,00	11,00	13,00
27	M	34	12,10	18,20	12,80	9,80	10,24	9,80	13,70	11,30	13,10
28	M	35	11,33	29,00	12,00	10,33	9,67	7,33	10,00	9,33	12,00
**Mean value**			12,9216,17	12,9231,14	12,9218,84	12,9214,96	12,9211,21	12,9211,65	12,9215,55	12,9210,85	12,9215,62
**SD**			12,928,19	12,9215,12	12,928,70	12,925,93	12,924,75	12,924,75	12,925,98	12,921,78	12,922,84
**Mann-Whitney U-test**			0,421348	0,118236	7,08E-05	0,00016501	1,58E-05	0,0229414	0,154337	0,535065	0,16807

Statistical analysis of methylation values for each CpG dinucleotide according to gestational age at birth.

Three CpG at 5’ extremity (N5, 6, 7) and one CpG at 3’ extremity (N28) of the analysed region showed a significantly higher methylation in preterm newborns using the nonparametric Mann-Whitney U-test.

P-values <0.05 are considered statistically significant.

Standard deviations are shown (SD).

M and F means male and female, respectively.

### Correlation of methylation profile with gestational age

**Fig 2:**
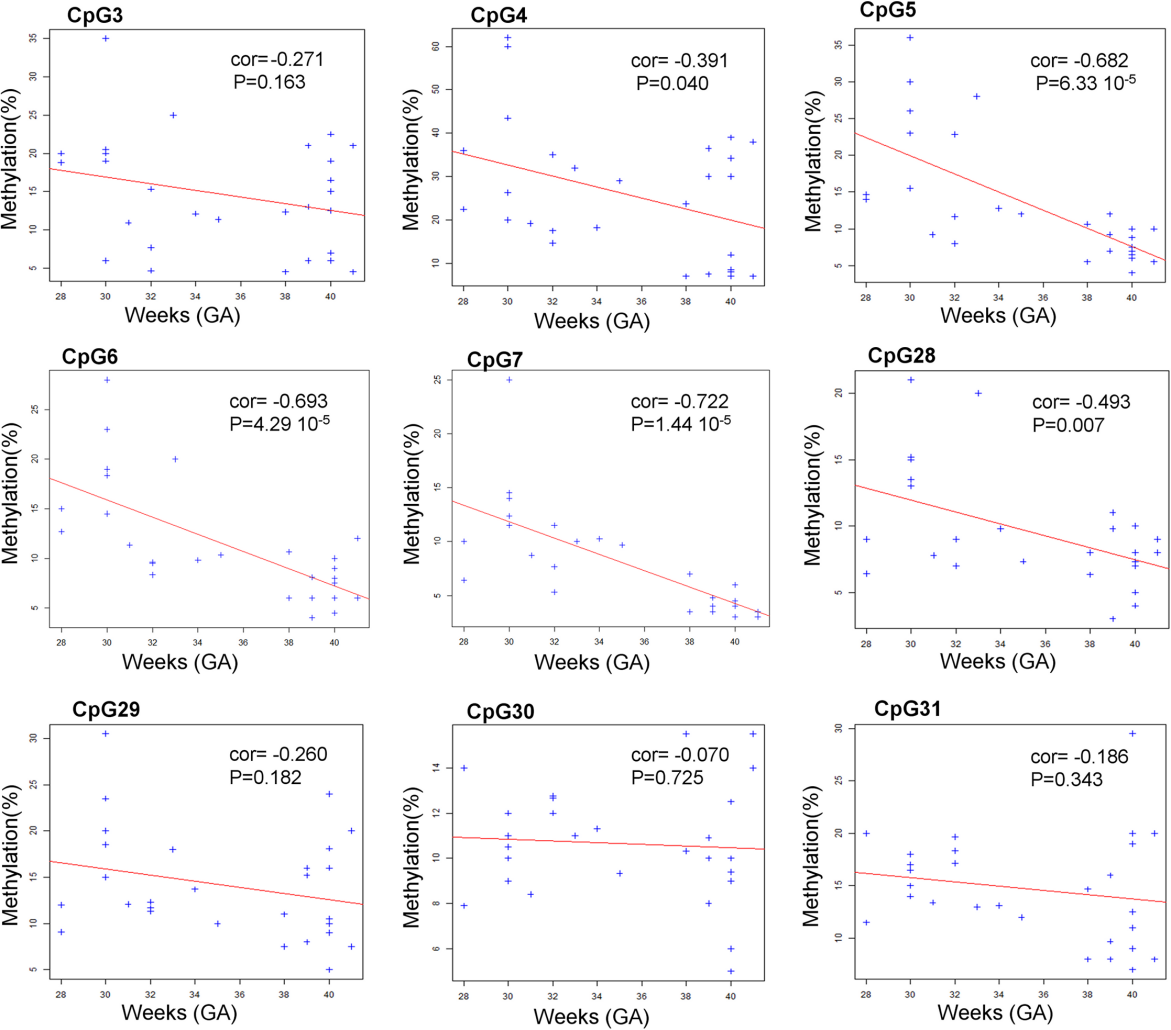
Correlation between methylation profile and gestational age. Bisulphite pyrosequencing results for each CpG dinucleotide of the *AMOT* promoter CpG island. Dots represent methylation values (y-axis) at each CpG for the corresponding gestational age in weeks (x-axis). We used this two variables as quantitative to estimate the relationship. The "cor" value for each plot represents the Pearson correlation coefficient that estimates the link between the two variables and P-values correspond to statistical relevance of the "cor" coefficient. The red lines correspond to the linear regression of the methylation profile as function of gestational age to illustrate the correlation in the plot. A negative value of the cor coefficient corresponds to a deacreasing of the methylation as function of the gestational age for the linear model (decreasing red line). Statistical were performed using R statistical tools.

We then analysed the relationship between the methylation level for each CpG dinucleotide and gestational age in weeks. The methylation level of CpG 4, 5, 6, 7 (at 5’ extremity) and the CpG 28 (at 3’ extremity) showed a negative correlation, statistically significant, with gestationnal age at birth (Fig 2).

In each CpG plot, the CpG dinucleotides have negative "cor" values that confirm that methylation deacreases when the gestational age increases. We have found relevant p-values for CpG 4, 5, 6, 7 (at 5' extremity) and the CpG 28 (at 3' extremity), but we can suppose that other CpG could be also relevant with more samples.

Altogether these results show a differential methylation in the *AMOT* CpG island associated with gestational age at birth.

### Expression of *AMOT* in ECFC

**Fig 3:**
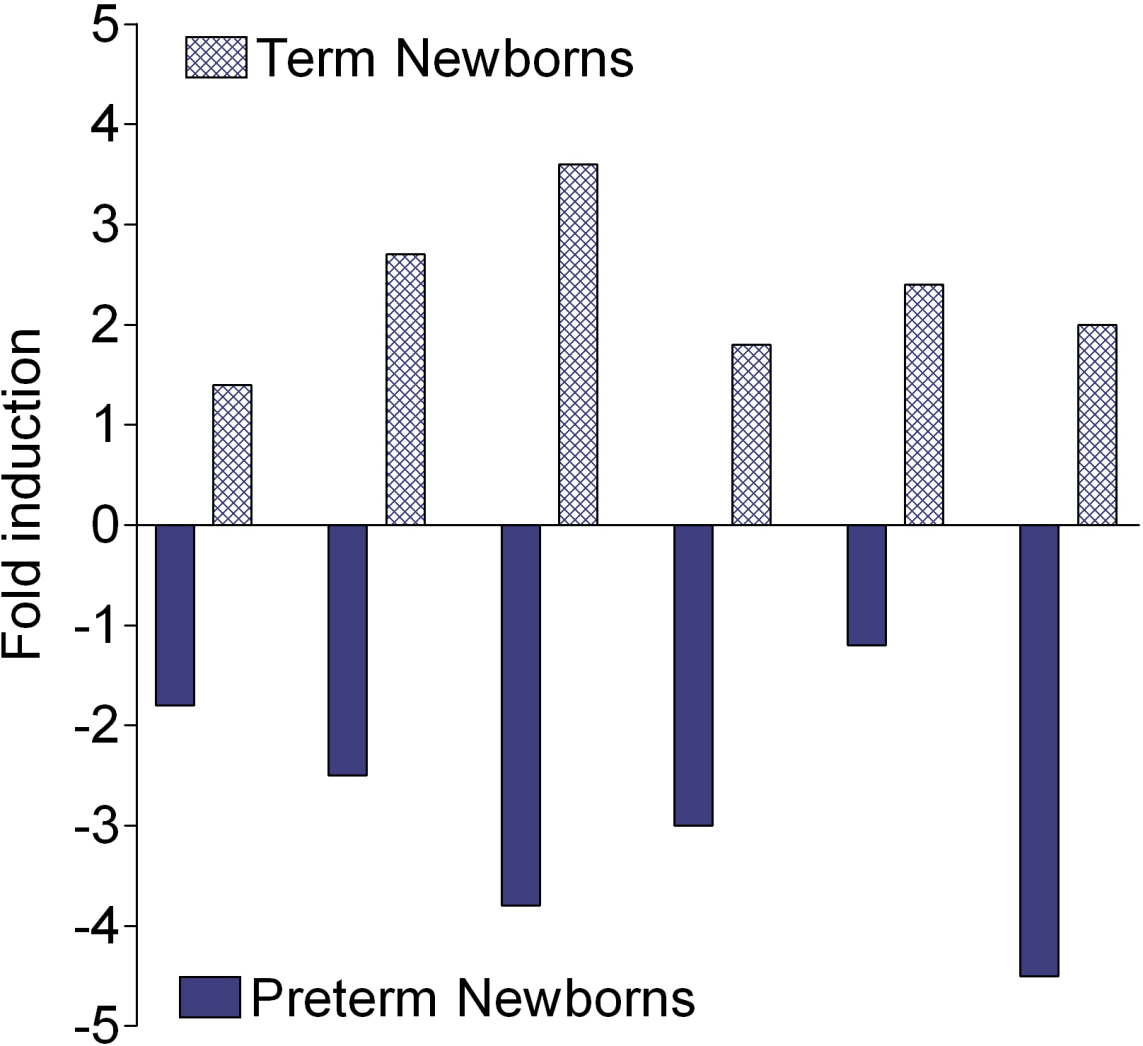
Modulation of *AMOT* gene expression. Changes in AMOT expression were assessed by qPCR on 6 cord blood ECFC from preterm neonates or their control counterparts. Each bar represents an individual sample. Each RNA from preterm ECFC was matched against an RNA from a control. Results were normalized to the values obtained with RPL13 expression.

The functional significance of the methylation profile of *AMOT* CpG island associated with preterm birth *per se* was analysed by qRT-PCR experiments. Analysis of 6 individual ECFC from preterm and term newborns, randomly chosen, showed a decreased expression of *AMOT* in all preterm newborns (Fig 3). These findings are in agreement with our previous results by real time-RT-PCR-array which identified forty-seven angiogenic genes linked to angiogenesis whose expression was modulated in preterm ECFC; in particular, the expression of *AMOT* was down-regulated (P-value 0.06055) [18]. This emphasizes that increased DNA methylation of *AMOT* is associated with the down-regulation of its expression.

## Discussion

The prenatal and perinatal periods are part of a unique period of sensitivity during early life, when «developmental plasticity» [21] allows adaptive responses to environmental stimuli which characteristically have not only short term effects, but can also have long-lasting consequences, due to altered developmental programming. These findings are the grounds of the Developmental Origins of Health and Disease concept (DOHaD), which underlines that preterm birth, in particular, is a risk factor for long-term non-communicable diseases, including hypertension [1,2].

Epigenetics plays an important role in embryonic and fetal development [22]. Furthermore, epigenetic changes also regulate angiogenic processes, through aberrant methylation in angiogenic genes. Hypermethylation of the anti-angiogenic factor thrombospondin 1 (*THBS-1*) and of *ADAMTS-8*, which has antiangiogenic properties, was found in different tumors [23–26]. Histone modifications also determine angiogenesis, regulating gene expression. Disruption of the deacetylase *Hdac7* gene expression in mice results in defective maintenance of vascular integrity during cardiovascular development [27]; disruption of *Sirt1* gene expression, which is another member of the histone deacetylase (*HDAC*) gene family, in zebrafish and mice results in defective blood vessel formation and ischaemia-induced neovascularization [28].

Evidence exists for epigenetic contributions to hypertension in animal models and identified different targets, namely: *i)* the upregulation of the angiotensin receptor gene (AT_1b_) in the rat adrenal gland by hypomethylation during the first week of life [29]; *ii)* an epigenetic regulation of the connective-tissue growth factor gene (CTFG) which involves the histone disruptor of telomeric silencing-1 (Dot1) and has a role in blood vessel remodeling and renal fibrosis [30,31]; *iii)* the enzyme chromodomain-helicase-DNA-binding protein 2 (CHD2) [32]. However, little information exists on the early epigenetic modifications of angiogenic genes in conditions involved in the developmental origins of hypertension.

We previously demonstrated that the expression of several genes that positively regulate angiogenesis was down-regulated in cord blood ECFC from preterm infants [18]. Among them, the *AMOT* gene is particularly interesting because it plays different crucial functions in the control of angiogenesis. The Amot protein is expressed as two different isoforms with distinct functions in angiogenesis. P80-Amot is expressed in endothelial cells and is involved in vascular tube formation, in cell migration *in vitro* and in endothelial cell polarization [33–35]. P130-Amot is expressed in various cell types, including vascular endothelial cells and is involved in the control of endothelial cell shape during the period of blood vessel stabilization-maturation [34,36]. In zebrafish, the Amot protein is crucial for endothelial migration during angiogenesis *in vivo* [37]; the *Amot* genetic knockdown severely inhibited migration of intersegmental vessels [19].

In mice, the *Amot* knockout resulted in 75% lethality around embryonic day 11, with severe vascular insufficiency in the intersomitic region and dilated vessels in brain [19]. In Amot-deficient endothelial cells the VEGF-induced tubulogenesis and endothelial cell polarization were impaired [19].

In the present study we focused on the methylation profile of the *AMOT* promoter CpG island during development, comparing it in ECFC of cord blood from term versus preterm newborns.

### Main discussion points

#### a) Preterm newborns have a significantly higher methylation profile of the *AMOT* promoter CpG island

We performed two kinds of experiments. Firstly, cloning-sequencing experiments were performed from cord blood ECFC of 3 preterm newborns (28 weeks GA) and 2 term newborns (>37 weeks GA) randomly chosen. Pyrosequencing experiments were subsequently realized from the ECFC of 14 preterm newborns at different gestational ages (ranging from 28 to 35 weeks GA) and 14 term newborns (>37 weeks GA).

The results of both cloning-sequencing and pyrosequencing experiments evidentiate that the *AMOT* CpG island had significantly higher level of methylation in the cord blood ECFC of preterm newborns than in the term newborns. No infants of diabetic mothers, a situation which impaired ECFC number and function [38], were included in this study. Our findings suggest that a direct relationship exists between preterm birth and the methylation level of *AMOT* in the ECFC of the cord blood.

Literature showed that genome-wide analysis of DNA methylation profile in umbilical cord blood from late preterm, term and post-term newborns (32–43 weeks GA) suggests variations of DNA methylation patterns across this range of gestational ages and evidences an association between the gestational age and a differential methylation of genes with a role in thyroid hormone synthesis, immune cell maturation, or apoptosis [39]; supplementary studies identified three differentially methylated regions which showed evidence for strong association between methylation and gestational age [40].

Altogether, these findings may help understanding the particular sensitivity of the early periods of life to altered environmental cues, with lifelong consequences.

#### b) The methylation profile of the *AMOT* promoter CpG island changes as a function of gestational age

The results of pyrosequencing experiments presented here showed a relationship between *AMOT* methylation level and gestational age: in fact, in five CpG dinucleotides the level of methylation decreased with the increasing of gestational age. As noted above, *AMOT* is a positive regulator of angiogenesis [19]; we speculated that the change in its methylation level with gestational age could be involved in a mechanism of developmental, temporal regulation of the angiogenic system and that, being born with a high methylation level of *AMOT*, may alter the on-going angiogenic process, which has to develop *ex utero*, in a completely different haemodynamic and homeostatic environment compared to the intrauterine one, would not preterm birth have occurred.

Of note, reduced EFCF number and function has been found as well and independently on gestational age, in infants of diabetic mothers, a condition which, similarly to preterm birth, leads to an increased risk of hypertension in adulthood [38]. This suggests that altered angiogenic characteristics in preterm infants are not solely a reflection of developmental immaturity. However, the influence of a progressively changing nutritional or stress environment of the fetus cannot be excluded.

Our study is performed on neonatal cord blood samples taken immediately after birth, not on fetal, intrauterine cord blood samples: the stress of delivery may have added effects to those of the developmental process. However, both preterm and term infants have been exposed to the stress of delivery, and it is unlikely that such stress may change according to gestational age, with the important amplitude observed in the preterm group.

Almost all neonates in the preterm group received antenatal steroid therapy to accelerate fetal maturation before birth. Steroid hormones are important mediators of fetal physiology and are involved in the developmental programming of the hypothalamic-pituitary-adrenal axis [11], with altered glucose tolerance and hypertension [41]. However, antenatal glucocorticoids induce genome-wide alterations oriented toward DNA hypermethylation [42], contrary to our observations.

In our preterm group antenatal exposure to exogenous steroids occurred at all gestational ages, as common practice, and cannot explain the observed gestational age-related methylation profile.

#### c) The methylation profile of the *AMOT* promoter CpG island is specific of the cord blood endothelial progenitor cells

Differences in methylation between cell types have been extensively reported for specific genes [43, 44]; however, attempts to sort tissues into subpopulations may limit the amount of DNA available for the study.

Our analysis was focused on the cord blood ECFCs; experiments using the cord blood mononuclear cells did not identify significant differential methylation in the two newborn populations (not shown). Thus, the *AMOT* methylation profile can be attributed to the specific endothelial cell lineage.

Previous results of our group demonstrated *in vitro* angiogenic defects of the cord blood ECFC in preterm newborns and established a link with changes in the angiogenic balance showing the upregulation of molecules with angiostatic properties (thrombospondins, PF4) and the down regulation of pregnancy specific angiogenic factors (PGF), or angiogenic specific factors: the *AMOT* expression was impaired [18]. This finding may be in correlation with the increased methylation status described here and is validated by the qRT-PCR results which showed that the *AMOT* expression is down-regulated in the ECFC of preterm newborns (Fig 3).

We can speculate that the methylation status of *AMOT* and its reduced gene expression may contribute, together with other angiogenic genes, to the anti-angiogenic state of ECFC in preterm newborns; in fact, we already evidentiated the up-regulation of the anti-angiogenic *THBS1* gene [18]. Furthermore, these analyses have been realized in the endothelial progenitor cells; consequently, the results may reflect the DNA methylation changes in the angio-system. These conditions can increase the risk of hypertension at adulthood.

### Perspectives

The findings of the present study may explain how the angio-epigenome can impair the function of preterm endothelial progenitors (ECFC), contributing to a supplemental susceptibility and an enhanced vulnerability to reach an optimal development of vascular networks.

The analysis was focused on the cord blood ECFCs; however, attempts to sort specific cells has limited the amount of DNA available for the study.

Short- and long-term studies are necessary to establish the chronological sequence of epigenetic changes: the follow-up of children born at term or prematurely could allow to determine how levels of methylation of the *AMOT* gene evolve *ex utero* in the two populations; the follow-up at adulthood will establish whether they develop arterial hypertension.

The DNA methylation analysis of other pro- and anti-angiogenic genes may identify genes involved in the long-term desease outcomes.

Otherwise, studies may be extended to the complications of preterm birth involving altered angiogenesis as a patho-physiologic mechanism, such as retinopathy of prematurity, or chronic lung disease.

Altogether, our results suggest that prematurity provides new insights into the link between specific epigenetic changes during development and mechanisms of long-term health outcomes.
